# Changes in the Microbial Community of the Mottled Skate (*Beringraja pulchra*) during Alkaline Fermentation

**DOI:** 10.4014/jmb.2003.03024

**Published:** 2020-05-08

**Authors:** Jongbin Park, Soo Jin Kim, Eun Bae Kim

**Affiliations:** 1 Department of Applied Animal Science, College of Animal Life Sciences, Kangwon National University, Chuncheon 24341, Republic of Korea; 2 Department of Animal Life Science, College of Animal Life Sciences, Kangwon National University, Chuncheon 24341, Republic of Korea

**Keywords:** *Beringraja pulchra*, alkaline fermentation, microbiome, *Pseudoalteromonas*, 16S rRNA gene

## Abstract

*Beringraja pulchra*, Cham-hong-eo in Korean, is a mottled skate which is belonging to the cartilaginous fish. Although this species is economically valuable in South Korea as an alkaline- fermented food, there are few microbial studies on such fermentation. Here, we analyzed microbial changes and pH before, during, and after fermentation and examined the effect of inoculation by a skin microbiota mixture on the skate fermentation (control vs. treatment). To analyze microbial community, the V4 regions of bacterial 16S rRNA genes from the skates were amplified, sequenced and analyzed. During the skate fermentation, pH and total number of marine bacteria increased in both groups, while microbial diversity decreased after fermentation. *Pseudomonas*, which was predominant in the initial skate, declined by fermentation (Day 0: 11.39 ± 5.52%; Day 20: 0.61 ± 0.9%), while the abundance of *Pseudoalteromonas* increased dramatically (Day 0: 1.42 ± 0.41%; Day 20: 64.92 ± 24.15%). From our co-occurrence analysis, the *Pseudoalteromonas* was positively correlated with Aerococcaceae (r = 0.638) and *Moraxella* (r = 0.474), which also increased with fermentation, and negatively correlated with *Pseudomonas* (r = -0.847) during fermentation. There are no critically significant differences between control and treatment. These results revealed that the alkaline fermentation of skates dramatically changed the microbiota, but the initial inoculation by a skin microbiota mixture didn’t show critical changes in the final microbial community. Our results extended understanding of microbial interactions and provided the new insights of microbial changes during alkaline fermentation.

## Introduction

*Beringraja pulchra* (mottled skate), a cartilaginous fish belonging to the family Rajidae, is found predominantly in the south sea of Korea, southern Japan, and East China Sea [1]. Skate have specific features that producing ammonia in their muscle during fermentation period in the low temperature [2]. During the fermentation, trimethylamine oxide (TMAO) and urea in the skate muscle are converted to trimethylamine (TMA) and ammonia, respectively [3]. Also, they produce mucus containing proteins, antibacterial enzymes, and proteolytic enzymes on their skins to prevent infectious pathogens and maintain innate immunity [4]. Certain strains of marine bacteria use these chemical compounds and producing antibacterial products which have probiotic potential found in the skate skin. Because of its specific features and flavors during fermentation, fermented skate is a popular seafood in South Korea and Iceland, with a high commercial value [5]. Especially, in Jeolla province of Korea, many consider it to be an irreplaceable food item for ceremonial occasions [6]. Continued skate ingestion can improve brain growth and cognitive function, alleviate symptoms of arthritis, improve skin health, prevent obesity, and it is also beneficial for the elderly [7, 8].

However, there are several safety concerns due to high concentrations of ammonia and bacterial contamination of fermented skate [9]. Thus, several studies on fermented skate have focused on the physicochemical and microbiological quality characteristics [10, 11]. According to a previous study, the pH of fermented skate ranged from 8.75–9.43 and the total number of microorganisms present in the skate was found to be between 4.8 log CFU/g and 7.5 log CFU/g [11]. In a study of prokaryotic community composition in alkaline fermented skate, the major phylum observed in the fermented skate was Firmicutes, whereas that in the fresh skate was Gammaproteobacteria [12]. However, only a small number of samples were analyzed and no repetition even using different conditions of fermentation period; therefore, limited information regarding the detailed bacterial distribution, interactions, or changes in microbial composition of the alkaline fermented skate is available. Furthermore, no previous studies have examined the effect of initial surface mucus microbiota on skate fermentation. Here, we investigated changes in the bacterial community composition in skate before, during, and after fermentation under different conditions such as the inoculation method (control vs. treatment) and effects of bacteria in different regions (skin & broth and flesh). Additionally, we examined the bacterial interaction networks to compare with previously investigated fish products.

## Materials and Methods

### Sample Preparation and Fermentation

**Fig. 1:**
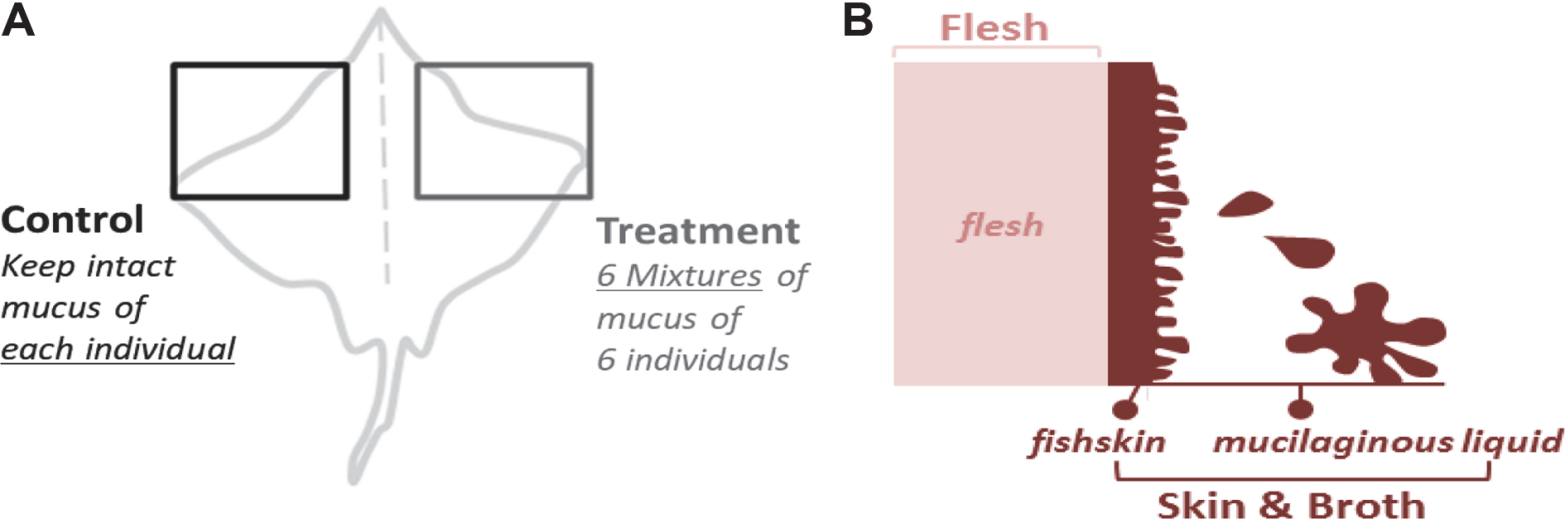
Sampling sites of skate during alkaline fermentation. (**A**) Control and treatment represent the difference based on the inoculation method, (**B**) Flesh and Skin & Broth represent different sampling sites from the skate body.

Six skates were captured around Daecheong island (Republic of Korea) by local fishermen, and samples were obtained with approval from the Institutional Animal Care and Use Committee at Kangwon National University (IACUC No.: KW-161010-2; Supplementary Table 1) [13]. All skates were preserved at -20°C during shipping before fermentation. After thawing, 11 skate wings were separated from six skates and fermented to compare the changes of microbiota at days 0, 10, and 20 of fermentation. The left wings (Control, *n* = 5) were fermented in their own individual “skin & broth,” while the right wings (Treatment, *n* = 6) were fermented with the skin and broth mixture of six skates (Fig. 1A). The flesh, as well as skin & broth regions of the skate were also compared before and after fermentation to investigate the effects of bacterial inoculation (Fig. 1B). All skate wings were stored in zipper bags (26.8 cm × 27.9 cm) at 4°C refrigerator for 20 days as a method for fermenting skate [12]. Samples were collected at days 0, 10, and 20 for pH and microbiological assessments.

### pH Measurements and Viable Cell Counts

During the fermentation period, skate samples were divided into 7-g portions, and 10-fold (v/w) sterilized water was added, followed by homogenization of the mixture at 10,000 rpm speed for 1 min with a homogenizer (Ultra Turrax T25 basic, Ika Werke Gmbh & Co., Germany) for measuring pH and cell counting. The homogenizer was thoroughly cleaned and washed three times with 70% ethanol (EtOH) before use. The pH was measured using a pH meter (720Aplus pH/ISE Meter; Thermo Orion) after homogenization. To calculate the total numbers of bacteria during the fermentation period, we inoculate the samples in several representative culture agar media. The homogenized samples were diluted up to six fold with 0.85% NaCl and plated onto several selective media; tryptic soy agar (TSA) for wide variety of bacteria, marine agar for marine bacteria, de man, rogosa, and sharpe agar (MRS) for *Lactobacilli*, violet red bile agar (VRBA) for coliform bacteria, and salmonella shigella Agar (SS) for *Salmonella* and *shigella* spp.. Plates were cultured at 37°C for 48 h and count viable cells (MB cell, Republic of Korea).

### DNA Extraction and PCR Amplification

Total genomic DNA was extracted from 250 mg of each homogenized sample using a NucleoSpin soil kit (Macherey-Nagel, Germany) according to the manufacturer’s protocol, and stored at −20°C until further analysis. The extracted genomic DNA was used as a template for a polymerase chain reaction (PCR), which was conducted to amplify 16S ribosomal RNA genes using barcoded primers targeting the V4 region. The V4 fragment of bacterial 16s rRNA gene is commonly used in microbial community analysis, because it provides sufficient phylogenetic richness for the gut and fermented food microbiota [14, 15]. PCR amplification was performed with Takara Ex-taq polymerase (Takara Bio, Japan) and 16S rRNA universal primers (Forward: 5′-GGACTACHVGGG TWTCTAAT-3′ and reverse: 5′-GTGCCAGCMGCCGCGGTAA-3′) using the following protocol: one cycle of 94°C for 3 min; 30 cycles of 94°C for 45 sec, 55°C for 1 min, and 72°C for 1.5 min; and one final cycle of 72°C for 10 min [16].

### Library Construction and Sequencing

For sequencing, size selection of adaptor-ligated DNAs and cleanup of PCR amplification were replaced by PCR product purification using a QIAquick PCR Purification Kit (Qiagen, USA). Libraries were constructed by C&K Genomics (Republic of Korea) and the constructed DNA libraries were confirmed by agarose gel electrophoresis; the amplicons were sequenced by Macrogen (Republic of Korea) using Illumina MiSeq platform.

### Microbial Community Analysis

Following skate fermentation, microbial communities were analyzed using Quantitative Insights Into Microbial Ecology (QIIME) version 1.9.1 (http://qiime.org) [17]. Raw reads were de-multiplexed and quality filtered using in-house perl scripts, then clustered into operational taxonomic units (OTUs) by closed-reference OTU picking at a 97% similarity using the GreenGenes 13_8 database [18]. Resulting BIOM-formatted file (http://biom-format.org/) were used for analyzing microbial diversity, taxa, and functional estimation. First, we tested α- diversity and β-diversity estimates. The α-diversity was determined using the richness estimators and diversity indices including Chao1, observed OTUs, phylogenetic diversity (PD), and Shannon index. These indices were calculated from 5,000 sequenced reads through rarefaction with ten iterations. OTUs were randomly selected at different reads in each sample (10, 509, 1008, 1507, 2006, 2505, 3004, 3503, 4002, 4501, and 5000). The β-diversity was calculated within QIIME using UniFrac distances among samples. Principal coordinate analysis (PCoA) was conducted based on unweighted and weighted UniFrac distances and visualized with EMPeror [19]. Relative abundance of microbial taxa was expressed as a percentage of the total 16S rRNA genes sequences at the phylum to the genus level. The relative abundance of phylum, family, and genus can be found in Supplementary Table 2, 3, and 4.

One-way analysis of variance (ANOVA) with Tukey’s post-hoc test using R (version 3.5.1) was used to identify significant differences during the skate fermentation.

### Metagenomic Estimation and Co-Occurrence Analysis

Functional genes from microbial communities were estimated using the Phylogenetic Investigation of Communities by Reconstruction of Unobserved States (PICRUSt) version 1.0.0 (http://picrust.github.io/picrust/) program [20]. BIOM-formatted files were normalized according to predicted 16S rRNA gene copy numbers, and predicted using precalculated Clusters of Orthologous Groups of proteins (COGs) and Kyoto Encyclopedia of Genes and Genomes (KEGG) pathways [21, 22]. Unclassified functional categories were removed from the analysis, and all tests to identify significant differences were two sided, with an indicating statistical significance (*p* < 0.05). To reveal the correlation among bacterial groups, we used the Spearman’s correlation coefficient using CoNet 1.1.1 [23], co-occurrence network inference analysis tool, and visualized using Cytoscape 3.7.1 [24].

### Statistical Analysis

All statistically analyzed samples were repeated more than three times using different skate samples. For statistics, Student’s *t-*test and one-way ANOVA with Tukey’s post-hoc test was performed to compare between groups or among groups, respectively. Less than 0.05 of *p* values were considered statistically significant.

### Omics Data

The raw 16s rRNA gene sequences data used in this study were deposited in the NCBI Sequence Read Archive (SRA) database with the SRA accession number PRJNA611462 (https://www.ncbi.nlm.nih.gov/sra/PRJNA611462).

## Results

### pH Measurements and Viable Cell Counts

**Fig. 2:**
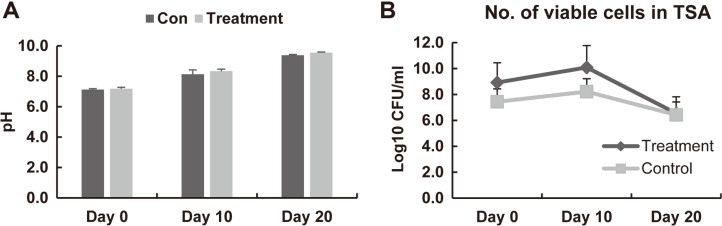
The pH value (A) and viable cell counts (B) during skate fermentation. Day 0: before fermentation; Day 10: during fermentation; Day 20: after fermentation; Control: left wings fermented with the skin & broth microbiota of each skate, Treatment: the right wings were inoculated with the skin & broth microbiota mixture obtained from six skates.

To examine the basic characteristics of the skates, we measured the pH and changes the number of viable cells during the fermentation (Fig. 2). The pH at days 0, 10, and 20 changed from 7.13, 8.13, and 9.39 in the control group, to 7.16, 8.34, and 9.54 in the treatment group, respectively (Fig. 2A). The pH increased from the beginning to the late stage of fermentation, but no significant difference was observed between the groups.

We just wanted to know whether such fermentation under low temperature is able to grow bacterial cells. So, changes in the number of viable cells were also confirmed as the pH changed during skate fermentation (Fig. 2B). Before fermentation, viable cells were detected on TSA, marine agar, and MRS agar, however VRBA and SS agar out of detectable range (Table 1). At day 10, higher numbers of viable cells were detected in TSA and marine agar compared to day 0, and colonies were detected on VRBA and SS agar. However, no colonies were detected within the detectable range in the MRS agar at day 10.

The number of viable cells increased on day 10 and decreased on day 20 in the TSA but this was not statistically significant. The number of viable cells on the marine agar, VRBA, and SS agar increased significantly at day 10. However, the number of cells in the SS and VRBA agar was decreased to the similar level as that on day 0 at day 20. There were no significant differences between the control and treatment samples.

### Microbial Diversities in Different Stages

**Fig. 3:**
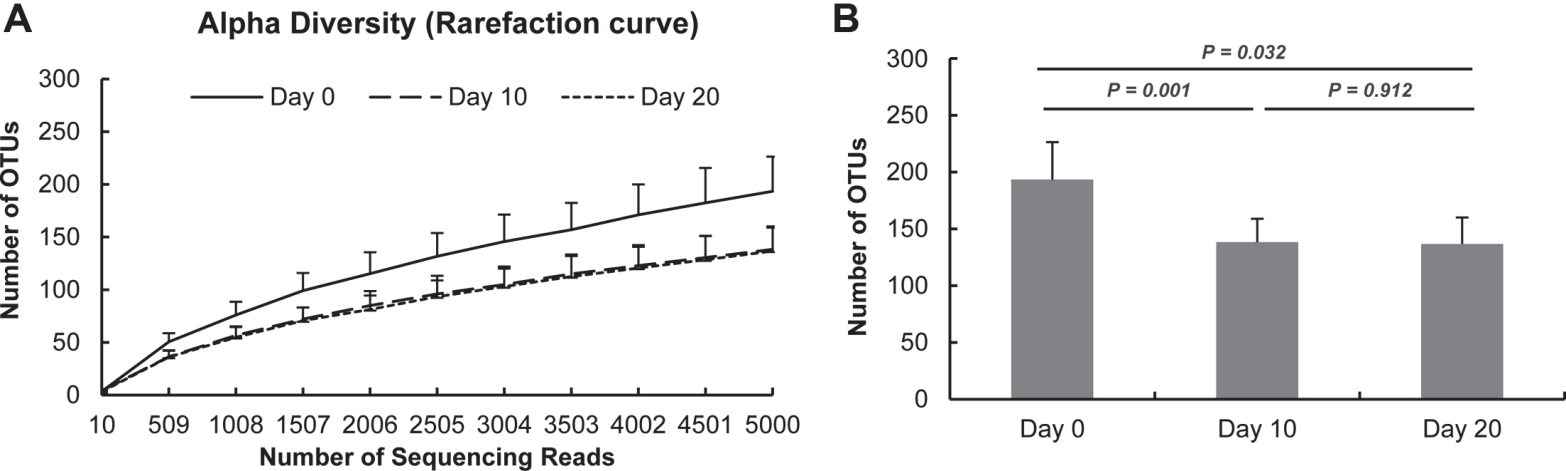
Comparison of microbial community diversity of skate before and after fermentation. Rarefaction curve (**A**) and Bar plot (**B**) showing observed OTU numbers at 5000 reads.

**Fig. 4:**
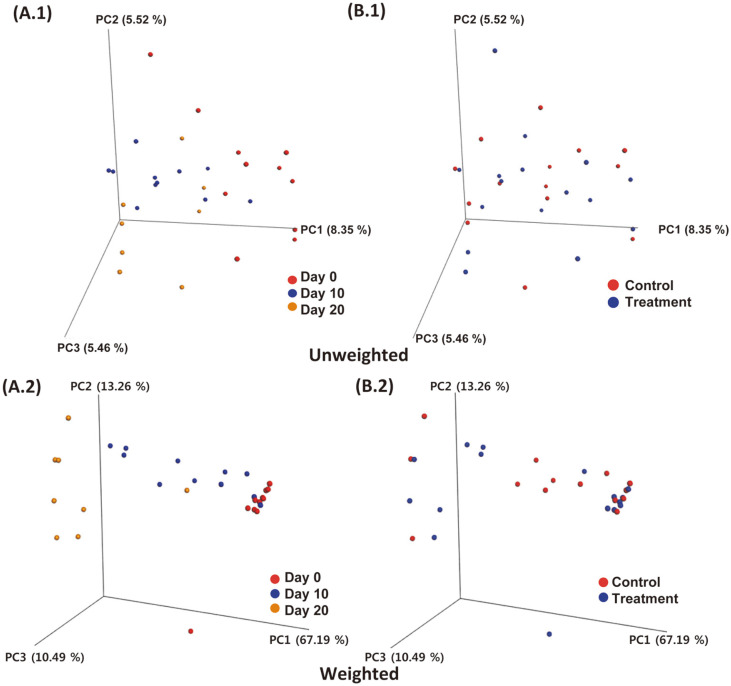
Principal coordinate analysis of unweighted and weighted plot based on UniFrac distance. Beta diversity patterns of skate samples based on the fermentation period (**A**), inoculation method (**B**), and different regions of the skate (**C**) were explored using principal coordinate analyses (PCoA).

From days 0 to 20, the diversity of the microbial community decreased gradually. The number of OTUs decreased significantly starting from day 10 (Fig. 3). However, there were no significant differences in the observed OTUs between day 10 and 20. The control and treatment groups were also compared, but there were no significant differences (data not shown). The levels of richness estimators, Chao1 and observed OTUs, were significantly higher at day 0 than those at days 10 and 20s (*p* < 0.05) (Table 2). PD value decreased significantly during this period. However, the Shannon index did not differ significantly during the fermentation period. Next, we analyzed the beta diversity of the skate during fermentation under three conditions (Fig. 4). Firstly, we compared the diversity during the fermentation period (Fig. 4A). In the unweighted plot, dots were scattered without the effects of the fermentation period (Fig. 4A.1). In the weighted plot, dots were clustered together according to their group except several dots (Fig. 4A.2). Specifically, dots were closely clustered at day 20. Secondly, PCoA plot was constructed according to the inoculation method (Fig. 4B). In both the unweighted and weighted plots, the control and treatment groups exhibited an overall distribution rather than forming distinct clusters. Thirdly, we investigated microbial diversity in different regions of the skate, i.e., flesh and skin & broth (Fig. S1A). In the unweighted plot, these regions formed different clusters on day 20 (Fig. S1, A2). In the weighted plot, the flesh and skin & broth clustered together on day 0 (Fig. S1, A3). However, clusters were grouped together on day 20 regardless of the sample type (Fig. S1, A4).

### Taxonomic Abundance of Fermented Skate During Fermentation Period

**Fig. 5:**
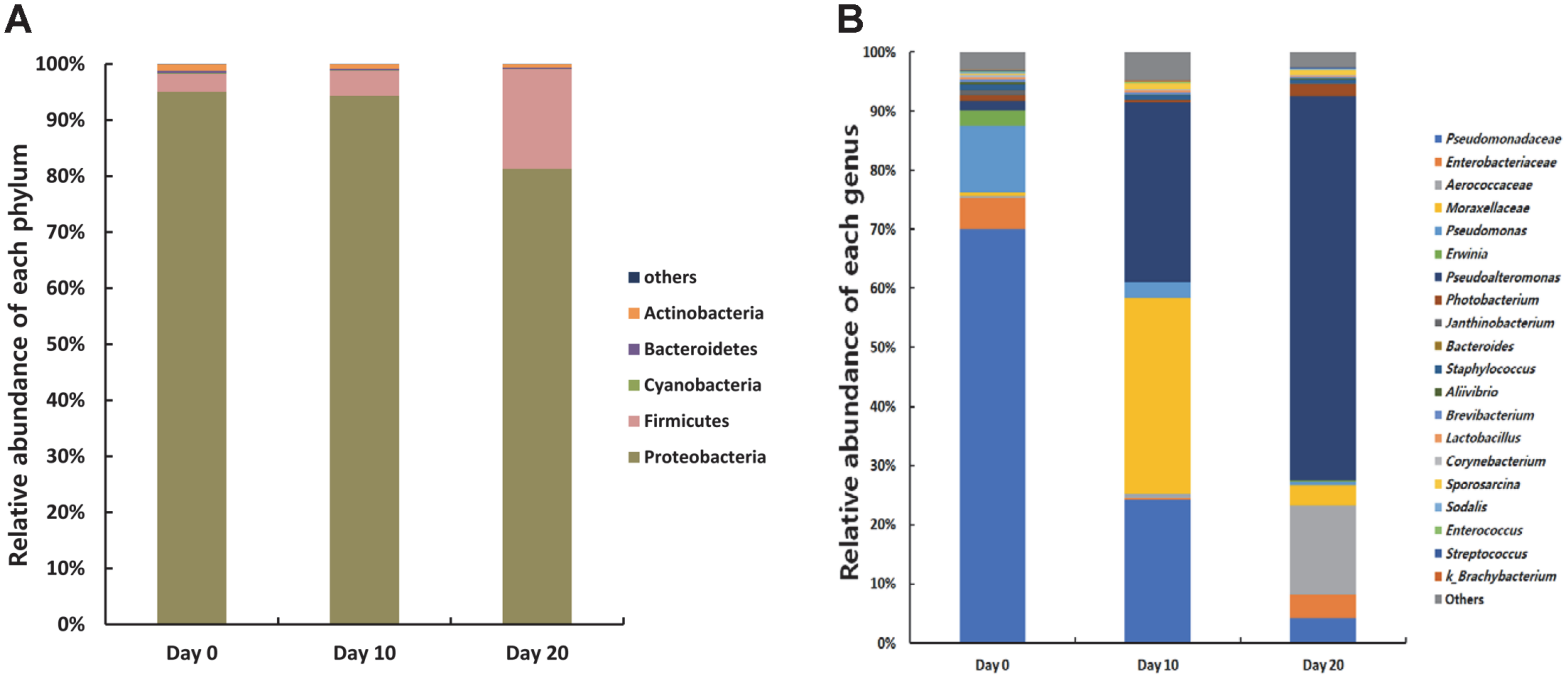
Relative abundance of bacterial community at phylum (A) and genus (B) level during fermentation period (Day 0, 10, 20).

We investigated the relative abundance of bacteria and archaea to trace bacterial changes during the fermentation period (Fig. 5). Each individual sample relative abundance showed in Supplementary Table 2, 3 and 4. Table 3 and 4 show only significantly different bacterial groups with over 0.01% bacterial composition. At day 0, the dominant bacterial phyla were Proteobacteria, followed by Firmicutes, Actinobacteria, Bacteroidetes, and Cyanobacteria. During fermentation, Proteobacteria significantly decreased (*p* < 0.001), but Firmicutes was significantly increase (*p* < 0.001; Table 3). In archaea, only Crenarchaeota and Euryarchaeota were found to be low percentages (< 0.01%).

At the family level, several groups such as Pseudomonadaceae, Pseudoalteromonadaceae, Moraxellaceae, and Aerococcaceae showed dramatic abundance changes during fermentation (Table 3). The predominant family was Pseudomonadaceae which constituted over 80% of bacteria in fermented skate at day 0. However, the abundance of Pseudomonadaceae decreased sharply after the fermentation period. In contrast, the abundance of the families Pseudoalteromonadaceae and Aerococcaceae increased markedly after the fermentation period. Interestingly, Moraxellaceae, was not a major family group at days 0 and 20, was predominant at day 10.

**Fig. 6:**
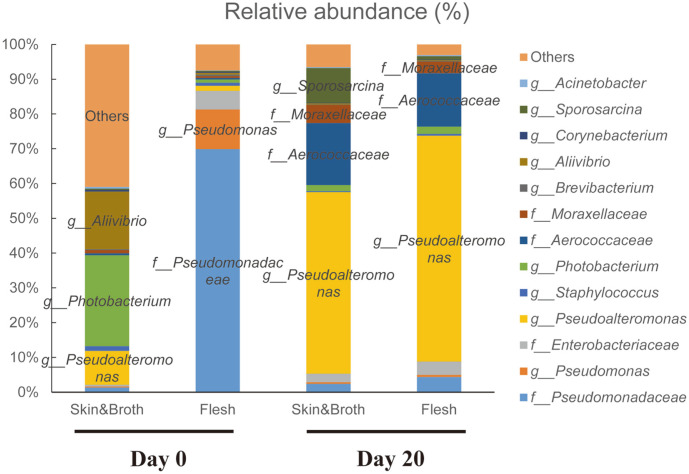
Relative abundance (%) of bacteria in ‘Skin&Broth’ and flesh on Day 0 and Day 20. Bacterial abundance ratio under 0.1% combine to others.

At the genus level, *Pseudomonas* and an unidentified genus, f__Pseudomonadaceae, which constituted a large proportion on day 0, decreased significantly at days 10 and 20 (Table 4). *Pseudoalteromonas*, which comprised a large proportion at day 20, significantly increased in abundance after fermentation (*p* < 0.001). The unidentified genus f__Moraxellaceae and *Psychrobacter* that belong to family Moraxellaceae were predominant only at day 10. The unidentified genus f__Aerococcaceae group was predominant at day 20. The abundance of lactic acid bacteria such as *Lactobacillus*, *Enterococcus*, and *Streptococcus*, which belong to the order Lactobacillales, decreased after fermentation but not significant. There are no significant differences between the control and treatment groups were observed. Pathogenic bacteria *Vibrio*, *Listeria, Salmonella,* and *Clostridium* were present only in a small proportion (< 0.01%) in the samples. We also compared bacterial composition of the skin & broth and flesh regions to evaluate the effect of the inoculum (Fig. 6). At day 0, the major genus of skin & broth region was *Photobacterium*, followed by *Allivibrio* and *Pseudoalteromonas*. The unclassified family Pseudomonadaceae and *Pseudomonas* were the most dominant in the flesh. After fermentation, both groups showed similar bacterial composition with no significant differences. *Pseudoalteromonas*, f_Aerococcaceae, and f__Moraxellaceae were the major genera in both the groups. On day 20, *Sporosarcina* (skin&broth: 10.016 ± 19.125%; flesh: 0.932 ± 0.966%) showed a higher abundance in the skin & broth group but no significant difference was observed owing to the high variation among the individual samples.

### Metagenomic Estimation of Skate Microbiota During Fermentation

We compared the COGs and KEGG pathways to predict for the functional and evolutionary microbiota of the skate during fermentation (Table 5 and 6). In COGs, “[E] Amino acid transport and metabolism”, and “[R] General function prediction only” were the major functional metabolism categories throughout the fermentation period (Table 5). All categories were the highest at day 0 and decreased significantly as the fermentation progressed. Before and after fermentation, COGs result of control and treatment groups (Day 0_Control vs. Treatment and Day 20_Control vs. Treatment) were not significantly different.

KEGG pathways in Supplementary Table 5, the overall pathway was also the highest at day 0 and decreased with fermentation except for the pathways listed in Table 7. “Transporter”, “ABC transporters”, and “DNA repair and recombination proteins” were the predominant pathways irrespective of the period (Table 6). Several pathways including “Phosphotransferase system (PTS)”, “Dioxin degradation”, “Xylene degradation”, “Bacterial toxins”, and “Protein digestion and absorption” pathways were significantly enriched over time (Table 7).

### Bacterial Networks during Fermentation Period

**Fig. 7:**
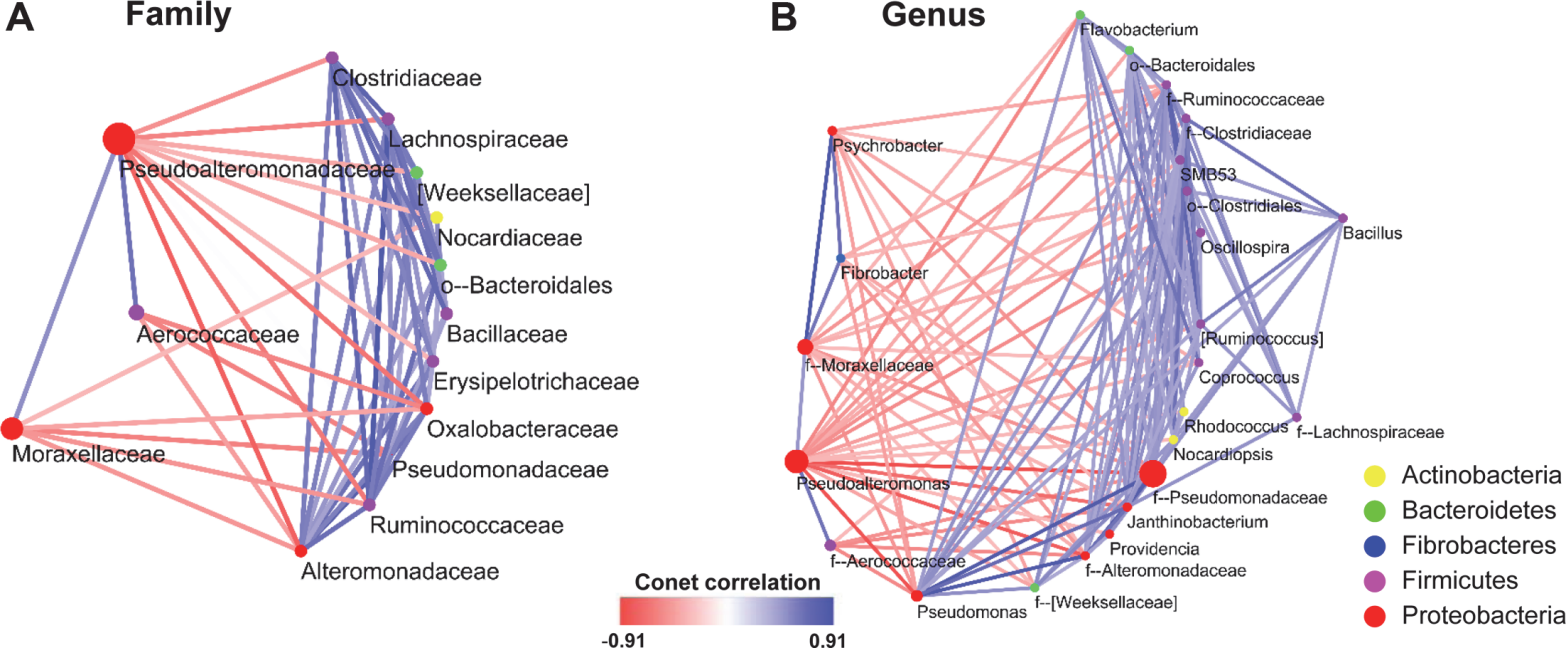
Conet co-occurrence network analysis during fermentation period. Each circle color represents taxonomic classification at the phylum level. Edge between the circle represents the correlation between each bacteria and transparency of the edge indicates the correlation score. Edge color indicates negative (red) and positive (blue) correlation, respectively.

To identify the network inferences between the bacteria during fermentation, we analyzed their co-occurrence using bacteria abundance data at the family and genus level (Fig. 7 and Table S6). At the family level, Pseudoalteromonadaceae was positively correlated with Aerococcaceae (Score = 0.644, *p* < 0.01) and Moraxellaceae (Score = 0.512, *p* < 0.01), while being negatively correlated with the rest of the bacterial families including Alteromonadaceae (Score = -0.767, *p* < 0.001), Oxalobacteraceae (Score = -0.666), and Clostridiaceae (Score = -0.519, *p* < 0.01). Moraxellaceae was negatively correlated with Oxalobacteraceae (Score = -0.429, *p* < 0.01), Pseudomonadaceae (Score = -0.596, *p* < 0.01), Ruminococcaceae (Score = -0.503, *p* < 0.01), and Alteromadaceae (Score = -0.493, *p* < 0.01). The correlation pattern of Aerococcaceae was similar to that of Ruminococcaceae, but not correlated. At the genus level, *Pseudoalteromonas* was positively correlated with the unidentified genus f-Aerococcaceae (Score = 0.637, *p* < 0.01) and f-Moraxellaceae (Score = 0.473, *p* < 0.01). The unidentified genus f-Moraxellaceae was correlated with *Psychrobacter* (Score = 0.906, *p* < 0.01) and *Fibrobacter* (Score = 0.622, *p* < 0.01).

## Discussion

In this study, we compared the microbial diversity, abundance, and bacterial correlation of skate during the fermentation period. Through our study, we confirmed that the biochemical trait and microbial diversity are influenced by the fermentation period. Skate specific product, skin & broth didn’t influence the microbial diversity and biochemical trait (pH). Moreover, the pH of skate changed from neutral to alkaline during the fermentation period and the pH condition was determined between day 10 or 20.

We used different agar media (TSA, Marine, MRS, VRBA, and SS) to confirm the changes in the number of viable cells and such fermentation under low temperature is able to grow bacterial cells. Marine agar primarily contains the minerals present in sea water such as sodium, magnesium, and calcium, which enriches the growth of certain marine bacteria such as *Vibrio*, *Pseudoalteromonas*, *Staphylococcus*, and *Pseudomonas* [25-27]. The number of viable cells in the marine agar increased during the fermentation period. Certain strains of marine bacteria may have survived or adapted to the high alkaline condition during the fermentation period. VRBA and SS agar which enrich pathogenic bacteria such as *Escherichia* spp., *Salmonella* spp., *Shigella* spp., and *Staphylococcus* spp. were significantly increased at day 10, but decreased on day 20. These bacterial groups are generally killed by high alkaline solution such as chlorine and ammonia in a wide pH range of 7.0–9.0 [28, 29]. We supposed that the increasing level of pH may influence the inhibition of certain bacteria species. However, several studies have reported that certain strains of these bacteria are tolerant to high alkaline conditions [30]. Therefore, the inhibition of these bacteria could be explained by the competitive inhibition between bacteria and pH condition. Further studies are required to reveal the relationships between the growth of these bacteria and pH condition.

Diversity indices and the number of OTUs significantly decreased at day 10 and remained constant until day 20. These results suggest that the microbial diversity of skate fermentation is determined at around day 10. Several studies have shown that the diversity of bacteria in fermented food or meat decreases at a specific time or stage according to acidic or alkaline conditions [15, 31].

Protein based fermented foods change their acid-base features, sensory properties, and microbial communities during fermentation at different storage temperatures or packaging conditions. In acidic fermented protein foods such as fermented milk and sausages, lactic acid bacteria (LAB) such as *Lactobacillus* spp., *Enterococcus* spp., *Pediococcus* spp., *Lactococcus* spp., and *Streptococcus* spp. are the predominant microorganisms after fermentation. These bacterial groups produce lactic acid, which prevents the growth of pathogenic bacteria and improves the flavor [15, 32, 33]. In alkaline fermented protein foods such as traditional eggs (Pidan) and soybean pastes (Doenjang, Gochujang, and Natto), *Bacillus* spp. and *Aspergillus* spp. are the major microorganisms [34]. Owing to their high proteolytic activity, these microorganisms use soybean as their protein source and produce nitrogen compounds [35]. Fermented cartilaginous fish, such as shark and skate contain urea and TMA N-oxide in their muscles which are distinct from meat muscle. These compounds are broken down during fermentation and produce high amounts of ammonia and TMA, resulting in alkaline conditions. Because of its various preservation methods, diverse bacterial communities are found after skate fermentation. In this study, the predominant family Pseudomonadaceae in the fresh skate transform to *Pseudoalteromonas* after fermentation. A previous study performed using fermented skate (*Dipturus batis*) skin and flesh showed conflicting results [36]. Both bacteria are frequently found in marine environment such as seawater, fish, shellfish, and marine algae [37, 38]. *Pseudomonas* are well-known gram-negative pathogenic bacteria that form biofilms and are resistant to antibiotics [39]. These bacteria are typically found on the skin and cause animal infections, ocean biofouling, and food contamination because of their pathogenic characteristics. *Pseudoalteromonas* is one of the most abundant taxonomic groups of the Proteobacteria found in the marine environment [40, 41]. *Pseudoalteromonas* spp. are frequently found in seafood such as Korean salted seafood (saeu-jeot), fermented brown shrimp, cod, and lobster [42, 43]. They may serve as probiotics because of their anti-pathogenic effects, as they produce bioactive compounds and are generally recognized as safe [41, 44]. They have the potential to break down protein, lipids, chitin in the fish by producing lipase, chitinase, amylase, agarase, and protease enzymes [42, 45]. Certain studies have reported that nitrogen regulates chitinase gene *chiA* of *Pseudoalteromonas* [46]. Previous studies have revealed that certain species of *Pseudomonas* can survive in a wide spectrum of pH ranging from 4.5 to 9.5 under refrigeration [47, 48]. Certain species of *Pseudoalteromonas* produce several antimicrobial metabolites that prevent the growth of gram-negative pathogens such as *Pseudomonas*, *Vibrio*, and *Escherichia* [49]. This suggests that the proportion change of *Pseudomonas* was more influenced by bacterial competitive inhibition rather than pH or preservation conditions.

*Psychrobacter* and family Moraxellaceae showed a rapid increase in abundance on day 10 and significant decrease on day 20, while the family Aerococcaceae showed the opposite trend on day 20. Certain genera of Moraxellaceae including *Psychrobacter* produce urease, such that they have the potential to degrade urea to ammonia and dioxide [9]. The abundance of certain *Psychrobacter* sp. increases when co-cultured with *Pseudoalteromonas* [42]. The co-occurrence analysis revealed that the family Pseudoalteromonadaceae positively correlated with Aerococcaceae and Moraxellaceae. Certain bacterial groups or high pH conditions on day 20 may influence the decrease in the abundance of the family Moraxellaceae. Aerococcaceae is a member of lactic acid bacteria found in fish and fermented food [50]. However, the role of these bacteria during fermentation has not yet been elucidated. In the current study, we showed that a new bacterial group could emerge even if a specific group of bacteria predominates.

Metabolic analysis revealed that the number of metabolic pathways decreased during fermentation in both COGs and KEGG. In COGs, “[R] General function prediction only” and “[E] Amino acid transport and metabolism” pathways were found to be predominant. “[E] Amino acid transport and metabolism” was more enriched than the carbohydrate and lipid metabolism pathways. KEGG analysis revealed that the major bacterial metabolic pathways were divided into two types of pathways. The first metabolic group including “transporter”, “abc transporters”, “DNA repair and recombination proteins”, “ribosome biogenesis”, “chromosome”, “secretion system,” and “bacterial motility proteins” may essential for the maintenance of bacteria in skate microbial population. The second group related to energy source utilization such as “purine metabolism”, “peptidases”, “amino acid related enzymes”, “valine, leucine, and isoleucine degradation”, “arginine and proline metabolism”, and “butanoate metabolism” may essential to microorganisms to use specific energy sources in the skate. The two groups of pathways revealed that certain bacteria may well adapted by theses metabolisms in the specific condition. Table 7 shows that several KEGG pathways are significantly enriched during fermentation. Energy source utilization pathways, such as “protein digestion and absorption”, “sphingolipid metabolism”, “glycosaminoglycan degradation”, “stilbenoid, diarylheptanoid and gingerol biosynthesis”, and “glycosphingolipid biosynthesis, globo series” were enriched during fermentation. Moreover, chemical compound degradation pathways such as “dioxin degradation”, “xylene degradation”, and “1,1,1-trichloro-2,2-bis(4-chlorophenyl) ethane (DDT) degradation” were significantly enriched. Skate may contain these compounds or may be affected by their contaminated habitat (Yellow sea) and utilized by bacteria. However, further studies, such as those analyzing the physicochemical properties and chemical compounds of skate in the sea, are required.

This study provided that the alkaline fermentation of skates dramatically changes the composition of microbiota, but the inoculation by a skin surface microbiota mixture didn’t affect the changes of final microbial community. The similarities and differences in bacterial composition during fermentation when compared to other studies were found as follows. Core bacterial groups (*Moraxella*, *Psychrobacter*, and *Pseudoalteromonas*) were also dominant as in other fish fermentations [43, 51]. Unlike other fermented seafood, a new bacterial family Aerococcaceae was detected. These findings provide us with better understanding of microbial communities during chilled fermentation and extended the new insights into microbial changes during alkaline fermentation.

## Supplemental Materials



Supplementary data for this paper are available on-line only at http://jmb.or.kr.

## Figures and Tables

**Table 1:** Total bacterial cells in different agar media.

Day	Day 0			Day 10			Day 20			*P^1^*

Agar type	Con	Treatment	Day 0.Average	Con	Treatment	Day 10.Average	Con	Treatment	Day 20.Average	
TSA	4.06±3.78	3.95±4.5	3.998±3.979	6.02±3.47	6.29±3.88	6.167±3.514	5±2.82	3.18±3.51	4.007±3.199	0.277
Marine	2.34±3.21	2.31±3.67	2.325±3.292^a^	7.87±0.59	6.34±1.41	7.038±1.333^b^	7.57±0.8	7.63±0.57	7.604±0.647^b^	0.000***
MRS	0±0	3.49±4.08	1.906±3.413	0±0	0±0	0±0	1.34±3	1.07±2.61	1.191±2.65	0.211
VRBA	0±0	0±0	0±0^a^	2.6±3.58	3.48±3.87	3.081±3.583^b^	0±0	1.07±2.61	0.582±1.929^a^	0.01*
SS	0±0	0±0	0±0^a^	4.05±3.7	2.82±4.37	3.376±3.926^b^	0±0	1.05±2.57	0.573±1.9^a^	0.008**

Data shown as the mean (log10 CFU/ml)±SD

^1^The P values were calculated using one-way ANOVA (* *P* < 0.05, ** *P* < 0.01; *** *P* < 0.001)

Different superscript letters (^abc^) indicate a significant difference (*P* <0.05) based on Tukey's post-hoc test.

**Table 2:** Microbial diversity indices during the fermentation period.

Item	Day 0	Day 10	Day 20	*P^1^*
Alpha diversity ^1^
Chao1	375.12±70.66^b^	308.41±60.76^ab^	269.36±53.27^a^	0.005^**^
Observed OTUs	185.38±35.3^b^	149.05±25.21^a^	133.8±22.58^a^	0.002^*^
PD	16.29±2.45^b^	12.98±2.02^a^	11.9±1.81^a^	0^**^
Shannon index	2.28±0.51	2.58±0.33	2±0.81	0.1

Data shown as the mean±SD.

^1^The P values were calculated using one-way ANOVA (*P < 0.05, **P < 0.01; *** *P* < 0.001)

Different superscript letters (^abc^) indicate a significant difference (*P* <0.05) based on Tukey's post-hoc test.

**Table 3:** Relative abundance of phylum and family during fermentation period.

Bacteria	Relative abundance (%)			*P^1^*

	Day 0	Day 10	Day 20	
Phylum				
Firmicutes	3.23±1.37^a^	4.51±2.79^a^	17.92±12.52^b^	< 0.001 ^* * *^
Proteobacteria	94.98±2.13^b^	94.31±3.11^b^	81.2±12.71^a^	< 0.001 ^* * *^
Cyanobacteria	0.14±0.08	0.09±0.06	0.06±0.04	0.059
Bacteroidetes	0.43±0.26	0.26±0.19	0.22±0.11	0.073
Actinobacteria	1.18±0.74	0.8±0.47	0.58±0.31	0.077
Family				
Pseudomonadaceae	81.29±11.44^b^	26.85±34.94^a^	4.98±7.43^a^	< 0.001 ^* * *^
Pseudoalteromonadaceae	1.43±0.42^a^	30.41±22.49^b^	64.92±24.15^c^	< 0.001 ^* * *^
Moraxellaceae	0.7±0.24^a^	35.27±23.47^b^	3.87±4.69^a^	< 0.001 ^* * *^
Aerococcaceae	0.47±0.22^a^	0.59±0.35^a^	15.38±12.33^b^	< 0.001 ^* * *^
Alteromonadaceae	0.04±0.01^b^	0.02±0.02^a^	0.01±0^a^	< 0.001 ^* * *^
Oxalobacteraceae	0.98±0.95^b^	0.05±0.08^a^	0.01±0.01^a^	0.001^* *^
Ruminococcaceae	0.13±0.08^b^	0.05±0.02^a^	0.04±0.02^a^	0.002^* *^
Lachnospiraceae	0.07±0.05^b^	0.04±0.02^a^	0.02±0.01^a^	0.002^* *^
o__Clostridiales;f__	0.04±0.02^b^	0.02±0.01^a^	0.02±0.01^a^	0.004^* *^
Clostridiaceae	0.06±0.04^b^	0.03±0.02^a^	0.02±0.01^a^	0.005^* *^
o__Bacteroidales;f__	0.04±0.04^b^	0.01±0.01^a^	0.01±0.01^ab^	0.022^*^
Erysipelotrichaceae	0.02±0.02^b^	0.01±0.01^ab^	0.01±0^a^	0.024^*^
Bacillaceae	0.13±0.08^a^	0.12±0.05^a^	0.06±0.03^a^	0.038^*^
f__[Weeksellaceae]	0.04±0.03^b^	0.02±0.02^ab^	0.01±0.01^a^	0.043^*^

^1^The P values were calculated using one-way ANOVA (*P < 0.05, **P < 0.01; *** *P* < 0.001)

Different superscript letters (^abc^) indicate a significant difference (*P* <0.05) based on Tukey's post-hoc test.

**Table 4:** Relative abundance of genus during fermentation period.

Genus	Relative abundance (%)			*P^1^*

	Day 0	Day 10	Day 20	
g__Pseudoalteromonas	1.42±0.41^a^	30.41±22.49^b^	64.92±24.15^c^	< 0.001 ^***^
f__Pseudomonadaceae;g__	69.9±9.9^b^	24.12±31.47^a^	4.36±6.54^a^	< 0.001 ^***^
g__Pseudomonas	11.39±5.52^b^	2.73±3.65^a^	0.61±0.9^a^	< 0.001 ^***^
f__Alteromonadaceae;g__	0.03±0.01^c^	0.02±0.01^b^	0±0^a^	< 0.001 ^***^
f__Moraxellaceae;g__	0.51±0.18^a^	33.09±22.08^b^	3.34±4.58^a^	< 0.001 ^***^
f__Aerococcaceae;g__	0.45±0.21^a^	0.58±0.35^a^	15.37±12.33^b^	< 0.001 ^***^
g__Psychrobacter	0.07±0.02^a^	1.92±1.61^b^	0.21±0.27^a^	< 0.001 ^***^
g__Janthinobacterium	0.9±0.85^b^	0.03±0.04^a^	0.01±0^a^	0.001^**^
f__Ruminococcaceae;g__	0.08±0.05^b^	0.03±0.01^a^	0.03±0.01^a^	0.002^**^
g__Coprococcus	0.01±0.01^b^	0±0^a^	0±0^a^	0.002^**^
g__Flavobacterium	0.01±0.01^b^	0±0^a^	0±0^a^	0.004^**^
o__Clostridiales;f__;g__	0.04±0.02^b^	0.02±0.01^a^	0.02±0.01^a^	0.004^**^
g__Rhodococcus	0.03±0.04^b^	0±0^a^	0.01±0.01^a^	0.007^**^
g__[Ruminococcus]	0.02±0.01^b^	0.01±0.01^a^	0.01±0.01^a^	0.011^*^
g__SMB53	0.04±0.03^b^	0.02±0.01^a^	0.01±0.01^a^	0.011^*^
g__Oscillospira	0.02±0.02^b^	0.01±0.01^a^	0.01±0.01^a^	0.011^*^
g__Providencia	0.05±0.04^ab^	0.08±0.05^b^	0.02±0.01^a^	0.017^*^
o__Flavobacteriales;f__[Weeksellaceae];g__	0.02±0.03^b^	0±0^a^	0.01±0.01^ab^	0.018^*^
o__Bacteroidales;f__;g__	0.04±0.04^b^	0.01±0.01^a^	0.01±0.01^ab^	0.022^*^
f__Lachnospiraceae;g__	0.02±0.02^b^	0.01±0^ab^	0.01±0^a^	0.024^*^
g__Bacillus	0.06±0.04^a^	0.06±0.03^a^	0.02±0.01^a^	0.040^*^
g__Nocardiopsis	0.04±0.05^a^	0.02±0.02^a^	0.01±0.01^a^	0.044^*^
f__Clostridiaceae;g__	0.02±0.01^b^	0.01±0.01^ab^	0±0^a^	0.047^*^

Data shown as the mean±SD.

^1^The P values were calculated using one-way ANOVA (* *P* < 0.05, ** *P* < 0.01; *** *P* < 0.001)

Different superscript letters (^abc^) indicate a significant difference (*P* <0.05) based on Tukey's post-hoc test.

**Table 5:** COGs pathways during fermentation period.

COGs	Relative abundance (%)			*P^1^*

	Day 0	Day 10	Day 20	
[A] RNA processing and modification	1335.9±123^c^	1027.5±174.3^b^	593.6±129^a^	< 0.001 ***
[B] Chromatin structure and dynamics	3163.4±408.5^c^	1859.7±864^b^	1088.9±154.4^a^	< 0.001 ***
[C] Energy production and conversion	291828±14182.5^c^	197029.5±58640.5^b^	125103±24797.5^a^	< 0.001 ***
[D] Cell cycle control, cell division, chromosome partitioning	44150.9±1775.6^c^	31767.6±7436.8^b^	25005.6±2364.4^a^	< 0.001 ***
[E] Amino acid transport and metabolism	420364±10576.5^c^	264519.4±85749.1^b^	182853.8±43896.2^a^	< 0.001 ***
[F] Nucleotide transport and metabolism	93928.5±2193^c^	67471.2±15356.1^b^	51975.3±8072.2^a^	< 0.001 ***
[G] Carbohydrate transport and metabolism	219911.9±15613.5^b^	111854.4±50117.1^a^	111545±36994.2^a^	< 0.001 ***
[H] Coenzyme transport and metabolism	187530.5±7404.8^c^	136530.9±31414.6^b^	87387.1±17007.7^a^	< 0.001 ***
[I] Lipid transport and metabolism	181937.9±11828.9^c^	126621.2±34423.3^b^	85179.4±10389.2^a^	< 0.001 ***
[J] Translation, ribosomal structure and biogenesis	212128.3±7324.4^c^	175333.8±25313.1^b^	136354.2±11426.9^a^	< 0.001 ***
[K] Transcription	350900.7±9419.8^b^	195807.3±83545.9^a^	159941.2±33056.4^a^	< 0.001 ***
[L] Replication, recombination and repair	236205.2±12356.5^c^	171817.2±42884.4^b^	120147.5±15703.6^a^	< 0.001 ***
[M] Cell wall membrane envelope biogenesis	242853.2±6796.2^c^	165637.9±43288.9^b^	122195±21195.9^a^	< 0.001 ***
[N] Cell motility	142146.9±10235.9^b^	73968.7±39921^a^	56596.1±12933.7^a^	< 0.001 ***
[O] Posttranslational modification, protein turnover, and chaperones	181348.3±9584.6^c^	130517.1±31667.2^b^	94051.9±11062.3^a^	< 0.001 ***
[P] Inorganic ion transport and metabolism	259953.4±6690.7^c^	164356.4±54298.7^b^	119439.2±23519.7^a^	< 0.001 ***
[Q] Secondary metabolites biosynthesis, transport, and catabolism	111825.2±6525.6^c^	65134.7±27099.7^b^	43064.1±8561.6^a^	< 0.001 ***
[R] General function prediction only	506324.7±16076.8^c^	325811.4±102077.8^b^	238086.2±43930.4^a^	< 0.001 ***
[S] Function unknown	392692.8±14029.6^c^	257921.4±76932.9^b^	193791±30569.5^a^	< 0.001 ***
[T] Signal transduction mechanisms	334372.5±24382^b^	182446.5±91147.8^a^	141828.4±19221.3^a^	< 0.001 ***
[U] Intracellular trafficking, secretion, and vesicular transport	124426.9±6196^c^	82995.2±23551.6^b^	60795.3±11593.6^a^	< 0.001 ***
[V] Defense mechanisms	66142.8±2527.6^c^	46172.5±12387.6^b^	36500.9±5348.5^a^	< 0.001 ***
[W] Extracellular structures	41±36.5	4.4±5.1	27.5±68.9	0.1476
[Z] Cytoskeleton	94.6±64^b^	21.1±25.8^a^	7±5.6^a^	< 0.001 ***

Data shown as average±SD.

^1^The P values were calculated using one-way ANOVA (*P < 0.05, **P < 0.01; ***P < 0.001)

Different superscript letters (^abc^) indicate a significant difference (*P* <0.05) based on Tukey's post-hoc test.

**Table 6:** Predominant KEGG pathways at level 3 during fermentation period.

Pathway level 3	Day 0	Day 10	Day 20	*P^1^*
Transporters	270807±14670.4^b^	140942.4±64764.9^a^	108633.1±49902.2^a^	< 0.001 ***
General function prediction only	184524±8071.4^b^	120480.1±38240.8^b^	89617.1±13657.7^a^	< 0.001 ***
ABC transporters	177844.9±8457^b^	94543.7±42620.4^a^	60310±33093.5^a^	< 0.001 ***
DNA repair and recombination proteins	105633.7±3765.7^c^	79443.5±16372.8^b^	62482.5±5873.4^a^	< 0.001 ***
Two-component system	144215.5±8180.7^b^	78699.6±37818.6^a^	59622.3±12999.7^a^	< 0.001 ***
Function unknown	106261.4±3721.2^c^	76558.8±17124.2^b^	61053.4±8261.5^a^	< 0.001 ***
Secretion system	106718.3±4525.1^b^	69364.3±20972.3^a^	54321.9±9556^a^	< 0.001 ***
Bacterial motility proteins	123051.8±10019.4^b^	65270±34585.3^a^	48490.6±10698.6^a^	< 0.001 ***
Purine metabolism	89462.5±3189.9^c^	64844±15132.5^b^	47995±6578.4^a^	< 0.001 ***
Ribosome	63906.1±2324^c^	55709.1±7035.7^b^	43104.6±4616.4^a^	< 0.001 ***
Ribosome Biogenesis	62343.7±2465.7^c^	50792.6±7522.5^b^	40928.5±2203.3^a^	< 0.001 ***
Other ion-coupled transporters	75147.9±2456.9^c^	48718.7±15455.4^b^	34251.8±8013^a^	< 0.001 ***
Chromosome	60822.7±1886.6^c^	47496.7±8345^b^	36069.9±3434.5^a^	< 0.001 ***
Transcription factors	79330±2645^b^	47068±16431.2^a^	42799.1±8630.9^a^	< 0.001 ***
Peptidases	61792.6±773.3^b^	46920.6±7770.1^a^	41774.4±3589.2^a^	< 0.001 ***
Arginine and proline metabolism	70411.1±3879.4^c^	46038.3±14568.3^b^	33319.9±4662.4^a^	< 0.001 ***
Valine, leucine and isoleucine degradation	56525.6±5738.3^c^	43045.2±10158.8^b^	26119.6±2849.8^a^	< 0.001 ***
Amino acid related enzymes	52295.1±1631.7^c^	42219.5±6850.5^b^	29191.5±3857.9^a^	< 0.001 ***
Pyrimidine metabolism	51354.2±1055.9^c^	41510.3±6280.9^b^	33682.9±3358.6^a^	< 0.001 ***
Butanoate metabolism	54546.7±3366.4^c^	40896.1±8984.3^b^	28422.6±3140.4^a^	< 0.001 ***

Data shown as average±SD.

^1^The P values were calculated using one-way ANOVA (* *P* < 0.05, ** *P* < 0.01; *** *P* < 0.001)

Different superscript letters (^abc^) indicate a significant difference (*P* <0.05) based on Tukey's post-hoc test.

Predominant pathway lists were calculated by the average of period (Day 0, Day 10, and Day 20) and only 20 pathways were listed in descending order.

Several pathways originated from human (*Homo sapiens*) were not shown in this study.

**Table 7:** Significantly increased KEGG pathways during fermentation period.

Pathway level 3	Day 0	Day 10	Day 20	*P^1^*
L3_Phosphotransferase system (PTS)	11278.4±3036.8^b^	4877.3±2818.8^a^	12909.1±5905.9^b^	< 0.001 ***
L3_Dioxin degradation	853.8±432.9^a^	972.2±510.2^a^	2452.6±316.6^b^	< 0.001 ***
L3_Xylene degradation	443.8±230.4^a^	701.7±402.4^a^	1740.9±322.7^b^	< 0.001 ***
L3_Bacterial toxins	791.8±173.7^a^	752.3±262.1^a^	1680.6±340.2^b^	< 0.001 ***
L3_Protein digestion and absorption	49.2±17.3^a^	384.5±275.7^b^	814.8±298.8^c^	< 0.001 ***
L3_Steroid hormone biosynthesis	207±64.8^a^	432.5±256.9^a^	825.5±290.3^b^	< 0.001 ***
L3_Lysosome	181.4±111.1^a^	299.6±152.9^a^	722.4±110.3^b^	< 0.001 ***
L3_Other glycan degradation	431.7±285.8^a^	379.7±173.1^a^	875.5±188.6^b^	< 0.001 ***
L3_1,1,1-Trichloro-2,2-bis(4-chlorophenyl) ethane (DDT) degradation	9.2±2.7^a^	186.9±137.7^b^	404.8±148^c^	< 0.001 ***
L3_Sphingolipid metabolism	487.4±236.4^a^	337±142.5^a^	810.6±150.6^b^	< 0.001 ***
L3_Ion channels	425.6±205.1^a^	302.3±117.4^a^	727.1±80.5^b^	< 0.001 ***
L3_Flavone and flavonol biosynthesis	18±7.6^a^	27±22.3^a^	199.8±152.7^b^	< 0.001 ***
L3_Glycosaminoglycan degradation	136±98^a^	86.5±43.6^a^	289.4±142.7^b^	< 0.001 ***
L3_Stilbenoid, diarylheptanoid and gingerol biosynthesis	65.3±21.9^a^	46.9±30.2^a^	213.8±155.1^b^	< 0.001 ***
L3_Glycosphingolipid biosynthesis, globo series	235.5±174.3^ab^	103.6±54.8^a^	354.8±212.5^b^	0.006 **
L3_Sporulation	445.1±190.1^a^	629.8±365.2^ab^	941±268.8^b^	0.005 **
L3_Nucleotide metabolism	622.7±577.8^ab^	307.1±141.4^a^	888.9±412.7^b^	0.018 *

Data shown as average±SD.

^1^The P values were calculated using one-way ANOVA (* *P* < 0.05, ** *P* < 0.01; ***P < 0.001)

Different superscript letters (^abc^) indicate a significant difference (*P* <0.05) based on Tukey's post-hoc test.

Only 17 pathways shown positive value (Day 20 - Day 0) were listed here.

Several pathways originated from human (Homo sapiens) were not shown in this study.
