# Supplementary material for: Changes in the Microbial Community of the Mottled Skate (Beringraja pulchra) during Alkaline Fermentation
Source: J Microbiol Biotechnol. 2020 May 8;30(8):1195–206. doi: 10.4014/jmb.2003.03024 (PMC9728196; doi:10.4014/jmb.2003.03024)
Supplement: Supplementary file 1 [file JMB-30-8-1195-supple.pdf]

J. Microbiol. Biotechnol.
https://doi.org/10.4014/jmb.2003.03024

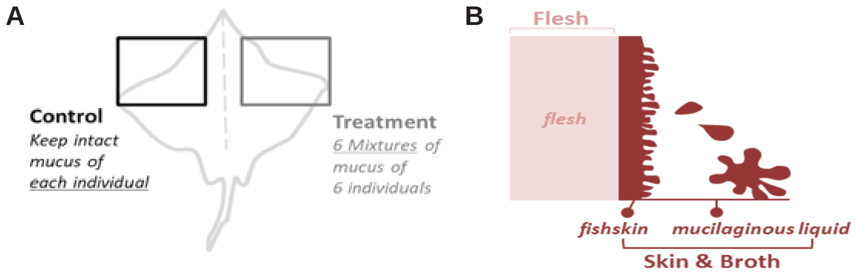


**Fig. 1.** Sampling sites of skate during alkaline fermentation. (A) Control and treatment represent the difference based on the inoculation method, (B) Flesh and Skin & Broth represent different sampling sites from the skate body.

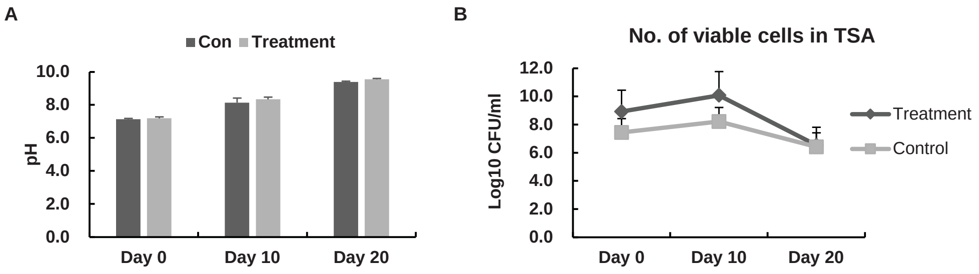


**Fig. 2.** The pH (A) and viable cell counts (B) during skate fermentation. Day 0: before fermentation; Day 10: during fermentation; Day 20: after fermentation; Control: left wings fermented with the skin & broth microbiota of each skate, Treatment: the right wings were inoculated with the skin & broth microbiota mixture obtained from six skates.

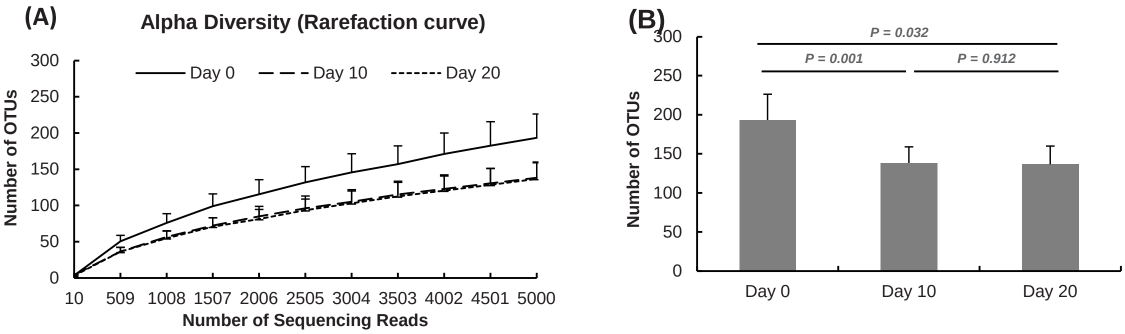


**Fig. 3.** Comparison of microbial community diversity of skate before and after fermentation. Rarefaction curve (A) and Bar plot (B) showing observed OTU numbers at 5000 reads.

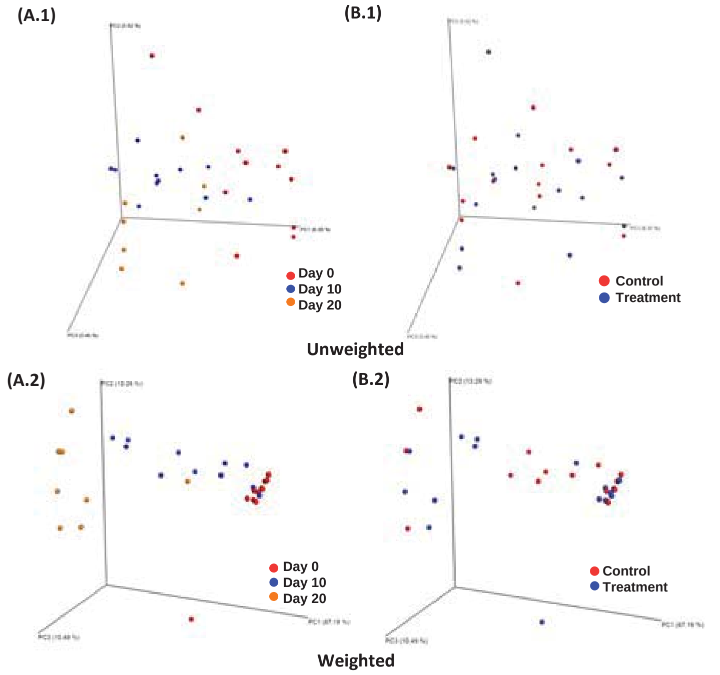


**Fig. 4.** Principal coordinate analysis of unweighted and weighted plot based on UniFrac distance. Beta diversity patterns of skate samples based on the fermentation period (A), inoculation method (B), and different regions of the skate (C) were explored using principal coordinate analyses (PCoA).

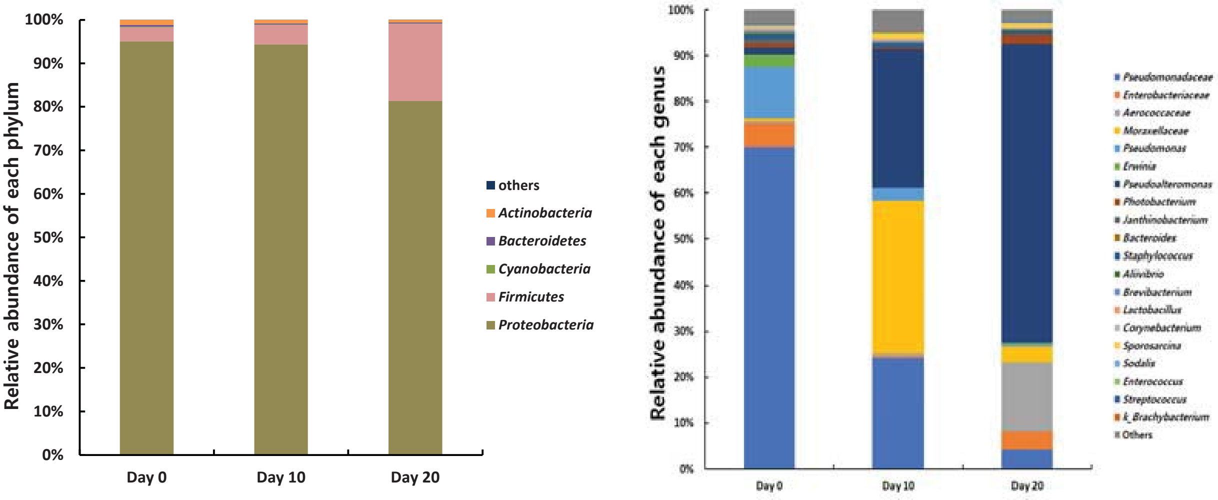


**Fig. 5.** Relative abundance of bacterial community at phylum (A) and genus (B) level during fermentation period (Day 0, 10, 20).

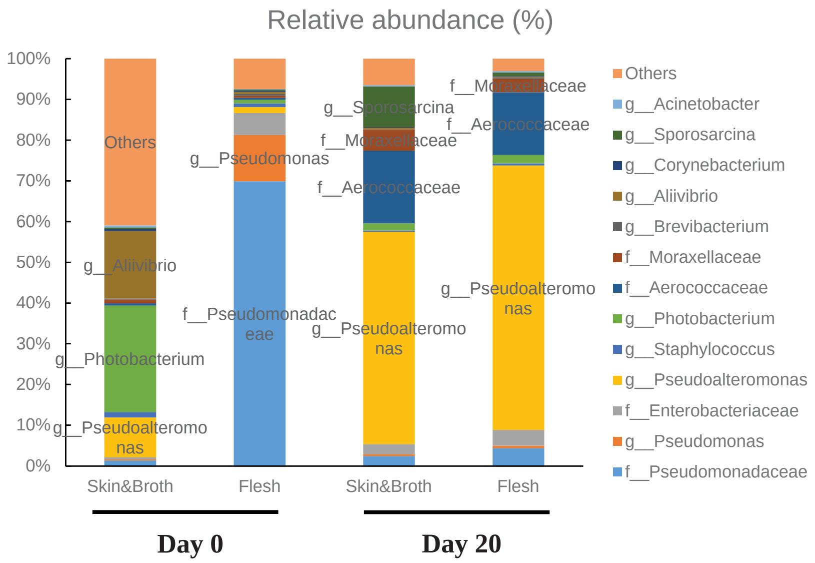


**Fig. 6.** Relative abundance (%) of bacteria in 'Skin&Broth' and flesh on Day 0 and Day 20. Bacterial abundance ratio under 0.1 % combine to others.

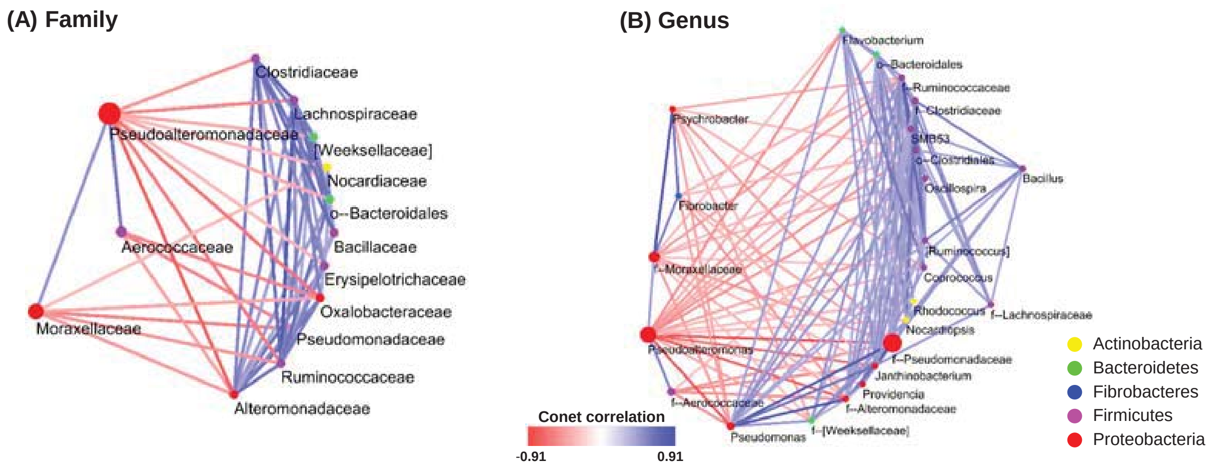


**Fig. 7.** Conet co-occurrence network analysis during fermentation period. Each circle color represents taxonomic classification at the phylum level. Edge between the circle represents the correlation between each bacteria and transparency of the edge indicates the correlation score. Edge color indicates negative (red) and positive (blue) correlation, respectively.
